# Peri-Hemorrhagic Edema and Secondary Hematoma Expansion after Intracerebral Hemorrhage: From Benchwork to Practical Aspects

**DOI:** 10.3389/fneur.2017.00004

**Published:** 2017-01-19

**Authors:** Marc-Alain Babi, Michael L. James

**Affiliations:** ^1^Division of Neuro-Critical Care, Department of Neurology, Duke University, Durham, NC, USA; ^2^Division of Neurosurgical Anesthesia, Department of Anesthesiology, Duke University, Durham, NC, USA

**Keywords:** ICH, intracerebral hemorrhage, brain injury, cerebral edema, intracranial pressure

## Abstract

Spontaneous intracerebral hemorrhage (SICH) is the most lethal type of stroke. Half of these deaths occur within the acute phase. Frequently observed deterioration during the acute phase is often due to rebleeding or peri-hematomal expansion. The exact pathogenesis that leads to rebleeding or peri-hemorrhagic edema remains under much controversy. Numerous trials have investigated potential predictor of peri-hemorrhagic edema formation or rebleeding but have yet to come with consistent results. Unfortunately, almost all of the “classical” approaches have failed to show a significant impact in regard of significant clinical outcome in randomized clinical trials. Current treatment strategies may remain “double-edged swords,” for inherent reasons to the pathophysiology of sICH. Therefore, the right balance and possibly the combination of current accepted strategies as well as the evaluation of future approaches seem urgent. This article reviews the role of disturbed autoregulation following SICH, surgical and non-surgical approaches in management of SICH, peri-hematoma edema, peri-hematoma expansion, and future therapeutic trends.

## Introduction

Spontaneous intracerebral hemorrhage (sICH) accounts for approximately 13–17% of all strokes; however, sICH carries substantial mortality and morbidity, approaching approximately 50% within 3 months and severe disability in the majority of survivors. Half of these deaths occur within the acute phase (1). Neurological deterioration during the acute phase may be due to hematoma expansion or peri-hemorrhagic edema growth (2). Since hematoma growth tends to occur within the first 24 h and edema formation within the first 72 h from symptoms onset, intervention during this time period may modify long-term outcome (2). Thus, the dynamic nature of early sICH represents a management challenge and opportunity for intervention. In this review, we discuss the pathogenesis and the role of different proposed pathways that have been explored to contribute to sICH progression.

## Pathogenesis

### Biology

The pathophysiology leading to hematoma expansion and edema progression remains poorly understood. sICH is believed to result from rupture of lipohyalinoic arteries followed by secondary arterial rupture at the periphery of the enlarging hematoma, in an “avalanche” fashion (2). This model was first proposed by C. Miller Fisher in the early 1970s (2, 3). Hematoma expansion may reflect additional leakage, extended spatial distribution of the initial hemorrhage, or both. Based on this model, mechanical disruption may be considered the most important neuropathological correlate for the expanding hematoma (2). Hematoma expansion leads to secondary injury mechanisms, which accentuates tissue destruction. Yet, exact pathophysiological mechanisms are unclear.

Prediction of risk factors for hematoma expansion and subsequent secondary injury might provide a first step toward development of effective therapies. Hematoma expansion and edema generation do not appear related to a single mechanistic pathway or risk factor, but rather several pathways/factors thought to act in synergy. Early preclinical models proposed the concept of “peri-hemorrhagic ischemia” surrounding the primary hematoma (2, 4–7). However, subsequent metabolism and flow studies demonstrated that such peri-hematoma changes were far from universal (7–10). Perihematomal changes lead to cytotoxic edema and neuroinflammatory mediators (11, 12).

### Role of Disturbed Inflammation

Numerous human and preclinical studies suggest a link between inflammation, peri-hematoma edema formation, and hematoma expansion. These studies particularly shed light on a direct role of neutrophil activation, free-radical formation, and the expression of interleukin-6 (IL-6) and tumor-necrosis alpha (TNF-α) (13–15). Several rat model studies have also shown that formation of the peri-hemorrhagic penumbra can be mediated by various neuroprotective elements such as *N*-methyl-d-aspartate receptor antagonism. The latter blunts excitatory amino acid-mediated neuronal death and diminishes microglia-mediated neuronal injury (11, 12, 16). Studies have also linked elevated plasma concentration of cellular fibronectin (c-FN) and inflammatory mediators IL-6 and TNF-α in the early phase of hematoma enlargement (13–15). However, the clinical utility of matrix metalloproteinase (MMP), c-FN, TNF-α, or IL-6 blood concentrations in early ICH remains unclear. Another distinct pathway that supports the role of neuro-inflammation in hematoma expansion includes thrombin-induced activation of inflammatory cascade; the latter being an important regulator of cellular activation through binding to the protease-activated receptors (PARs) expressed on platelets, leukocytes, and endothelial cells (ECs) (17–20), along overexpression of MMP (17–19). The latter promotes extracellular matrix proteolysis, attack the basal lamina, and results in degradation of c-Fn (17–19, 21). The expression of such inflammatory processes seem to coincide chronologically with the peak of peri-hemorrhagic edema formation and secondary hematoma expansion; when its maximal potential is often reached by 3–5 days from the initial ictus of hematoma formation (2, 10, 22, 23).

### Role of Disturbed Autoregulation

Disturbed autoregulation and uncontrolled perfusion pressure in hypertension may act as a driving force for hematoma expansion and peri-hemorrhagic edema formation. Numerous studies have suggested that blood pressure elevation may worsen ICH by providing continued force for hematoma expansion and potentially worsening outcomes (24, 25). However, aggressively blood pressure lowering after sICH may be counterintuitive. Elevation in mean arterial pressure may be a natural response to preserve cerebral perfusion. Qureshi et al. (26) describe three distinct phases of metabolic changes with respect to autoregulation: hibernation, seen during the first 48 h with reduction of CBF, and metabolism occurring in bilateral cerebral hemispheres; reperfusion, which may last up to 14 days with heterogeneous areas of cerebral hypo- and hyperperfusion; and finally, normalization, with resolution and development of normal cerebral flow pattern except in non-viable brain tissue (3, 26–32). Numerous models demonstrated that acute blood pressure reduction is associated with decreased diffusion on brain imaging (21, 33). However, studies have found no clear clinical implication of these findings (34, 35). Major randomized clinical trials (ATACH, INTERACT, and INTERACT-2) have explored the relationship of blood pressure reduction and clinical outcomes in ICH. While no sustained long term outcome benefit has been found for aggressive blood pressure management, interventions do appear to be safe (36–38). More recently, the ATACH 2 trial further re-affirmed that intense BP control (target 110–139 mmHg) did not result in an incremental benefit or lower rate of death or disability than standard reduction to a target of 140–179 mmHg (21, 33–35, 39).

### Role of Hemostasis

While homeostatic therapies seem promising, through prevention of hematoma enlargement, clinical trials examining use of blood products (in particular recombinant factor VIIa) remains inconclusive. While initial preliminary data suggested that Factor VIIa may be safe (40, 41), results from a phase-3 randomized controlled trial showed that although recombinant factor VIIa use after ICH resulted in significant reduction in hematoma volume but no reduction in severe disability or death compared to placebo at 3 months (42). If fact, recombinant factor VIIa use after ICH was associated with higher risk of arterial thromboembolic adverse events (43). The current AHA/ASA guidelines have since concluded that recombinant factor VIIa remains investigational and should not be used in sICH (44). While there is no disagreement in regard of coagulopathy reversal for patients’ who develop acute intracerebral hemorrhage while on anticoagulant therapy, the role of platelet transfusion remains controversial. A recent multicenter randomized controlled trial suggested (PATCH) suggested that platelet transfusion is inferior to standard of care for patients who develop intracerebral hemorrhage while on antiplatelet therapies, and thus cannot be recommended (45).

### Surgical Hematoma Evacuation

Surgical evacuation of the hematoma, and on whether this is beneficial, remains under investigation. Under select circumstances, various surgical approaches may be undertaken. This may include conventional craniotomy, stereotactic guidance with aspiration and thrombolysis, image-guided stereotactic endoscopic aspiration, and decompressive craniectomy. The overall aim of surgical intervention is to remove the source of hemorrhage, eliminate the localized or global mass effect of the hematoma, and eliminate the toxic effects of blood degradation products. To date, two major randomized controlled trials (STITCH I and STICH-II) explored surgical vs non-surgical management of ICH (46, 47). However, those trials failed to show an outcome benefit over conservative treatment. However, one of the largest meta-analysis which also included the STICH-II data suggested an overall benefit for surgery for select subgroups of patients, including those with poorer prognosis at presentation, those with secondary deterioration attributed to hematoma expansion, and those with superficial ICH without intraventricular extension (48).

Recently, minimally invasive and stereotactic surgeries have emerged as an alternative to craniotomy for hematoma evacuation. The more recently published ICES (intraoperative stereotactic computed tomography-guided endoscopic surgery) study suggested that early computerized tomographic image-guided endoscopic surgery is a safe and effective method in select cases to remove acute intracerebral hematomas, with a potential to enhance neurological recovery (49). Similarly, the MISTIE trial (minimally invasive surgery plus alteplase) in intracerebral hemorrhage evacuation appeared overall safe and promising in ICH (50). However, many questions remain regarding the surgical optimization of the endoscopic technique, the patients’ selection, and the timing of surgery. The role of minimally and endoscopic surgery will continue to evolve as more centers continue to gain experience with this promising approach.

## Conclusion

Current treatment strategies may remain “double-edged swords.” For example; surgical intervention may reduce hematoma volume but may also lead to decompression of the surrounding “peri-hemorrhagic penumbra tissue” with subsequent re-accumulation of bleeding. Likewise, hemostasis might stop cerebral bleeding yet compromise normal circulation. Blood pressure reduction decreases hematoma expansion but may also decrease cerebral perfusion and other vital organ blood flow. Therefore, balance of current accepted strategies and the evaluation of future approaches seem critical. This topic will continue to evolve as our understanding of the pathogenesis of sICH and secondary hematoma expansion continue to evolve.

## Author Contributions

M-AB and MJ contributed to the preparation and drafting of this manuscript. All authors have read and approved this manuscript in its final form.

## Conflict of Interest Statement

The authors declare that the research was conducted in the absence of any commercial or financial relationships that could be construed as a potential conflict of interest.
